# Revealing Public Opinion Towards COVID-19 Vaccines With Twitter Data in the United States: Spatiotemporal Perspective

**DOI:** 10.2196/30854

**Published:** 2021-09-10

**Authors:** Tao Hu, Siqin Wang, Wei Luo, Mengxi Zhang, Xiao Huang, Yingwei Yan, Regina Liu, Kelly Ly, Viraj Kacker, Bing She, Zhenlong Li

**Affiliations:** 1 Department of Geography Oklahoma State University Stillwater, OK United States; 2 Center for Geographic Analysis Harvard University Cambridge, MA United States; 3 School of Earth and Environmental Sciences University of Queensland Brisbane Australia; 4 Department of Geography National University of Singapore Singapore Singapore; 5 Department of Nutrition and Health Science Ball State University Muncie, IN United States; 6 Department of Geosciences University of Arkansas Fayetteville, AR United States; 7 Department of Biology Mercer University Macon, GA United States; 8 Department of Computer Science University of Massachusetts Lowell Lowell, MA United States; 9 College of Computing Georgia Institute of Technology Atlanta, GA United States; 10 Institute for Social Research University of Michigan Ann Arbor, MI United States; 11 Geoinformation and Big Data Research Laboratory Department of Geography University of South Carolina Columbia, SC United States

**Keywords:** Twitter, public opinion, COVID-19 vaccines, sentiment analysis, emotion analysis, topic modeling, COVID-19

## Abstract

**Background:**

The COVID-19 pandemic has imposed a large, initially uncontrollable, public health crisis both in the United States and across the world, with experts looking to vaccines as the ultimate mechanism of defense. The development and deployment of COVID-19 vaccines have been rapidly advancing via global efforts. Hence, it is crucial for governments, public health officials, and policy makers to understand public attitudes and opinions towards vaccines, such that effective interventions and educational campaigns can be designed to promote vaccine acceptance.

**Objective:**

The aim of this study was to investigate public opinion and perception on COVID-19 vaccines in the United States. We investigated the spatiotemporal trends of public sentiment and emotion towards COVID-19 vaccines and analyzed how such trends relate to popular topics found on Twitter.

**Methods:**

We collected over 300,000 geotagged tweets in the United States from March 1, 2020 to February 28, 2021. We examined the spatiotemporal patterns of public sentiment and emotion over time at both national and state scales and identified 3 phases along the pandemic timeline with sharp changes in public sentiment and emotion. Using sentiment analysis, emotion analysis (with cloud mapping of keywords), and topic modeling, we further identified 11 key events and major topics as the potential drivers to such changes.

**Results:**

An increasing trend in positive sentiment in conjunction with a decrease in negative sentiment were generally observed in most states, reflecting the rising confidence and anticipation of the public towards vaccines. The overall tendency of the 8 types of emotion implies that the public trusts and anticipates the vaccine. This is accompanied by a mixture of fear, sadness, and anger. Critical social or international events or announcements by political leaders and authorities may have potential impacts on public opinion towards vaccines. These factors help identify underlying themes and validate insights from the analysis.

**Conclusions:**

The analyses of near real-time social media big data benefit public health authorities by enabling them to monitor public attitudes and opinions towards vaccine-related information in a geo-aware manner, address the concerns of vaccine skeptics, and promote the confidence that individuals within a certain region or community have towards vaccines.

## Introduction

As of May 21, 2021, the COVID-19 pandemic had led to more than 160 million confirmed cases and more than 3 million deaths worldwide [1]. COVID-19 has continued to spread worldwide due to its highly contagious nature, diverse variants, and the mass public’s inconsistent adherence to effective public health measures, such as wearing masks and maintaining social distance [2]. Meanwhile, the emergence of asymptomatic cases (which are difficult to detect) has become more frequent, potentially leading to a substantial accumulation in the number of infections over time [3]. As such, it is important to keep COVID-19 vaccines widely available and accessible [4].

Since January 2020, scientists and medical experts around the world have been developing and testing COVID-19 vaccines; 16 vaccines have been approved for emergency use around the world so far, but the progress of vaccination has been subject to hesitancy, distrust, and debate. Vaccine hesitancy was identified by the World Health Organization as one of the top 10 global health threats in 2019 [5]. In many countries, such hesitancy, along with vaccine misinformation, have presented substantial obstacles towards vaccinating a sufficient amount of the population in order to establish herd immunity [6,7].

Therefore, it is crucial for governments, public health officials, and policy makers to understand the potential drivers that affect public opinion towards COVID-19 vaccines [8]. A number of campaigns against antivaccination activists have been made through multiple channels since January 2020. Notably, the accelerated pace of vaccine development has further heightened public anxieties and could compromise the public’s acceptance of the vaccine [9]. However, this acceptance varies across geographic contexts and the pandemic timeline. As governments put more effort into developing strategies for promoting vaccine acceptance and uptake, the key questions regarding the willingness to be vaccinated persist — what are the public’s opinions and perceptions towards COVID-19 vaccines and what are the potential drivers that affect such opinions?

The internet and social media have provided rich user-generated data sources, in the form of infodemiology studies [10], in real time for performing public health surveillance [11]. Social media, especially Twitter, have been considered as major channels for the distribution of health information and opinion exchange, helping people to make intelligent decisions [12,13]. The analysis of big data derived from Twitter has been an emerging trend in recent COVID-19 vaccine–related studies. Geotagged tweets (hereinafter termed as geotweets) provide a rich volume of cost-effective content, including news, events, public comments, and the locational information of Twitter users. Through sentiment analysis and topic modeling methods that have been widely used in existing studies, qualitative tweet contents can be retrieved to reflect public opinions and attitudes towards COVID-19 vaccines. Additionally, users’ location information enables researchers to investigate the spatiotemporal patterns of the public’s opinions and attitudes. In general, existing studies have investigated people’s reactions towards COVID-19 vaccines, with a geographical emphasis on the United States [14-19]. Some papers have also studied other countries in the world, including China [20], South Africa [21], Australia [22], the United Kingdom [14,23], Canada [24], and Africa [25], and to a global scale [26]. However, the study period of these works is relatively limited to or predominantly focused on the early stage of the pandemic or up to the end of 2020. None of these studies cover early 2021, the period of implementing mass systemic vaccine distribution. Furthermore, although sentiment analysis and topic modeling have been broadly applied, what remains less explored are the potential drivers that induce a change in public sentiment and opinion on vaccines, such as important events and announcements by political leaders (eg, the propaganda of vaccine success or vaccine conspiracy theories). There is a pressing need to investigate public opinion towards COVID-19 vaccines across a longer timeline and to explore the potential drivers that influence the change in such opinion over time.

To address these knowledge gaps, this study aimed to analyze the spatiotemporal patterns of public sentiment and emotion and explore the keywords and major topics of tweets regarding COVID-19 vaccines that were tweeted by Twitter users. Drawing on more than 300,000 geotweets from March 1, 2020 to February 28, 2021 in the United States, we employed sentiment and emotion analysis at both the national and state levels. We identified 3 phases along the pandemic timeline that display sharp changes in public sentiment and emotion. Using cloud mapping of keywords and topic modeling, we identified 11 key events and major topics as the potential drivers that induced such changes. Findings from this study can help governments, policymakers, and public health officials understand factors that motivate and cause hesitance in the public towards vaccination. With this understanding, these entities can better design potential interventions during their vaccination campaigns.

## Methods

### Data

Using the Twitter streaming Application Programming Interface (API), the Harvard Center for Geographic Analysis collected geotweets from March 1, 2020 to February 28, 2021. Geotweets provide the location information of user-defined places. If users activate the GPS function in Twitter, their longitude and latitude are provided. We used the keyword “vaccin*” to query vaccine-related tweets, generating a total of 308,755 geotweets. In the results, 1.43% (44,118/308,755) of geotweets’ geographic locations are at a state level (ie, Massachusetts, United States), and others are geocoded at a city level (ie, Cambridge, MA) or at a finer geographical level (ie, Uptown Coffee, Oxford, MS). We then conducted a series of data preprocessing of the geotweets’ contents. First, we generalized the variations of COVID-related terms to “COVID-19,” including “corona,” “covid,” “covid19,” and “coronavirus”; second, we removed unrelated website links from the search results, including links starting with the fragment of “https”; third, we removed punctuation (eg, period, question mark, comma, colon, and ellipsis) and other key symbols (eg, bracket, single and double quotes) and converted capital letters into lower-case letters; fourth, we removed inflectional endings (eg, “ly”) and reverted words to their root or dictionary form (eg, “peopl” from people, “dai” from daily, and “viru” from virus), by employing the *word lemmatization* function provided in the Python package Natural Language Toolkit 3.6.2 [27].

### Methodology

To explore the spatiotemporal patterns of public sentiment and emotion towards COVID-19 vaccines, we conducted 4 sets of analyses, including sentiment analysis, emotion analysis, topic modeling, and word cloud mapping. For the sentiment analysis, we applied Valence Aware Dictionary for Sentiment Reasoning (VADER), a well-known rule-based model, to estimate sentiment compound scores [28]. The sentiment compound score is computed by summing the score of each word in the lexicon, adjusted according to the rules. The rules embody grammatical and syntactical conventions for expressing and emphasizing sentiment intensity. Then, the score is normalized to be between –1 (most extreme negative) and +1 (most extreme positive). To reclassify sentences as positive, neutral, or negative sentiment, threshold values are set as follows: A tweet with a compound score larger than 0.05 is classified as positive sentiment; a tweet with a compound score smaller than –0.05 is classified as negative sentiment; otherwise, it is classified as neutral sentiment [28]. We then cross-tabulated the 3 types of sentiment on daily and weekly bases with the number of geotweets. We generated line graphs at the national level and in the top 10 states with the largest number of geotweets.

Different from sentiment analysis, which detects positive, neutral, or negative feelings from tweet contents, emotion analysis aims to recognize the types of feelings more specifically through the content expression, such as anger, fear, and happiness. The emotion analysis of this study was performed based on the National Research Council Canada Lexicon (NRCLex) [29]. NRCLex examines 4 pairs of primary bipolar emotions: joy (feeling happy) versus sadness (feeling sad); anger (feeling angry) versus fear (feeling of being afraid); trust (stronger admiration and weaker acceptance) versus disgust (feeling something is wrong or nasty); and surprise (being unprepared for something) versus anticipation (looking forward positively to something). We then examined the temporal patterns of these 8 types of emotion at both national and state levels.

In order to investigate the potential drivers of such changes, we applied the Latent Dirichlet Allocation (LDA) model [30] to detect popular topics based on a certain number of key dates as the turning points of sentiment scores or with a sharp change in the number of geotweets. The LDA model generates automatic summaries of topics in terms of a discrete probability distribution over words for each topic and further infers per-document discrete distributions over topics [31]. Each topic is treated as a cluster, and each document is assigned to a cluster that represents its dominant topic. LDA is an unsupervised algorithm [32], meaning that, prior to running the model, users need to predefine the number of topics. To estimate the optimal number of topics, we used the Python package [33] and pyLDAvis [34] to compare the results with topic numbers from 3 to 10 and found that the smallest overlap among topics occurs when the topic number is 3. We further visualized the topic modeling results in bar graphs with the Y-axis, which indicates the top 10 keywords associated with that topic, and the X-axis, which shows the weight of each keyword (to reveal the extent to which a certain keyword contributes to that topic). Based on the top 10 most relevant keywords to each topic, we generalized and presented the name of each topic at the bottom of each graph.

We then categorized the study period into 3 phases based on 2 iconic events: the results of Phase 1 clinical trials by Moderna that were published in *The New England Journal of Medicine* on July 14, 2020 [35] and the first COVID-19 vaccine shots that were given in the United States on December 14, 2020 [36]. Phase 1, dating from March 1, 2020 to July 13, 2020, is the stage in which the public was waiting for official announcements regarding the effectiveness of COVID-19 vaccines; Phase 2, ranging from July 14, 2020 to December 13, 2020, is when the positive news of COVID-19 vaccine development began to arrive; and Phase 3 starts from December 14, 2020, when the first vaccine shots were given in the United States. We then aggregated sentiment scores at the state level and analyzed the changes in sentiment over the 3 phases in the top 10 states. Finally, we produced word cloud maps over the 3 predefined phases based on the frequency of keywords appearing in Tweet contents, with the size of a keyword reflecting its frequency and popularity.

## Results

### Sentiment Analysis and Topic Modeling

**Figure 1:**
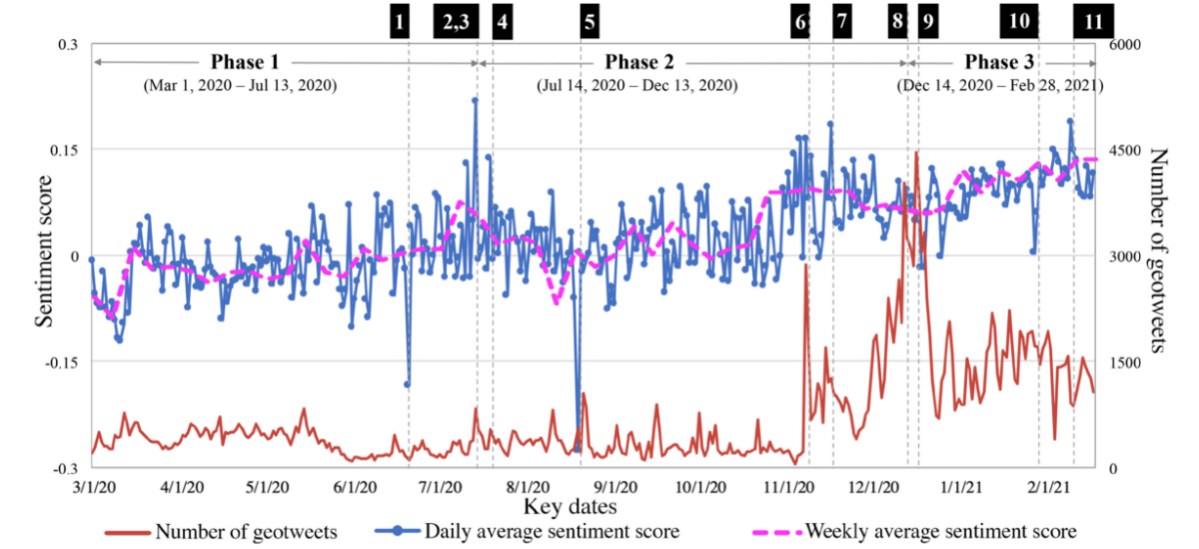
Sentiment scores and the number of geotweets over the entire study timeline at the national level.

**Figure 2:**
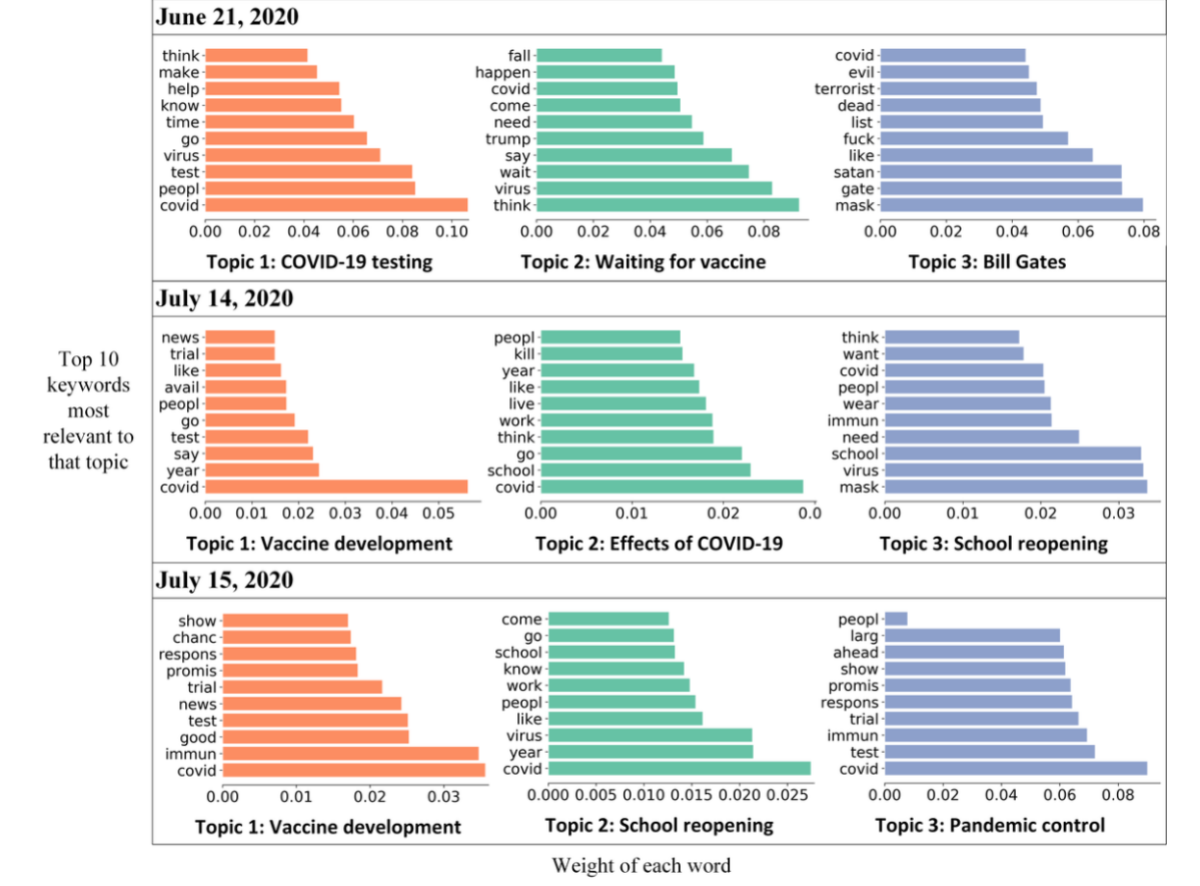
Three topics discussed on each of 3 key dates: June 21, 2020; July 14, 2020; and July 15, 2020.

**Figure 3:**
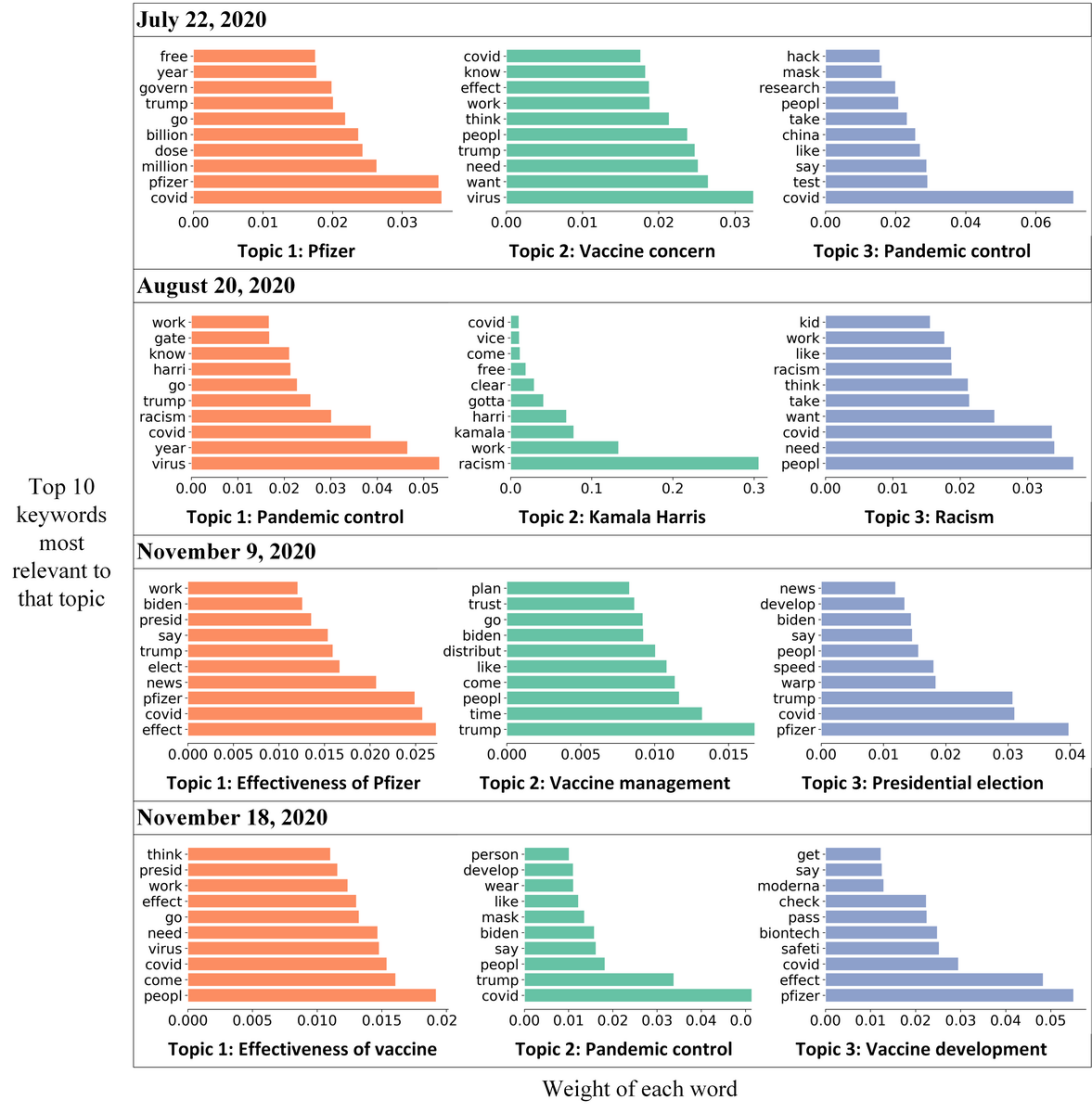
Three topics discussed on each of 4 key dates: July 22, 2020; August 20, 2020; November 9, 2020; and November 18, 2020.

**Figure 4:**
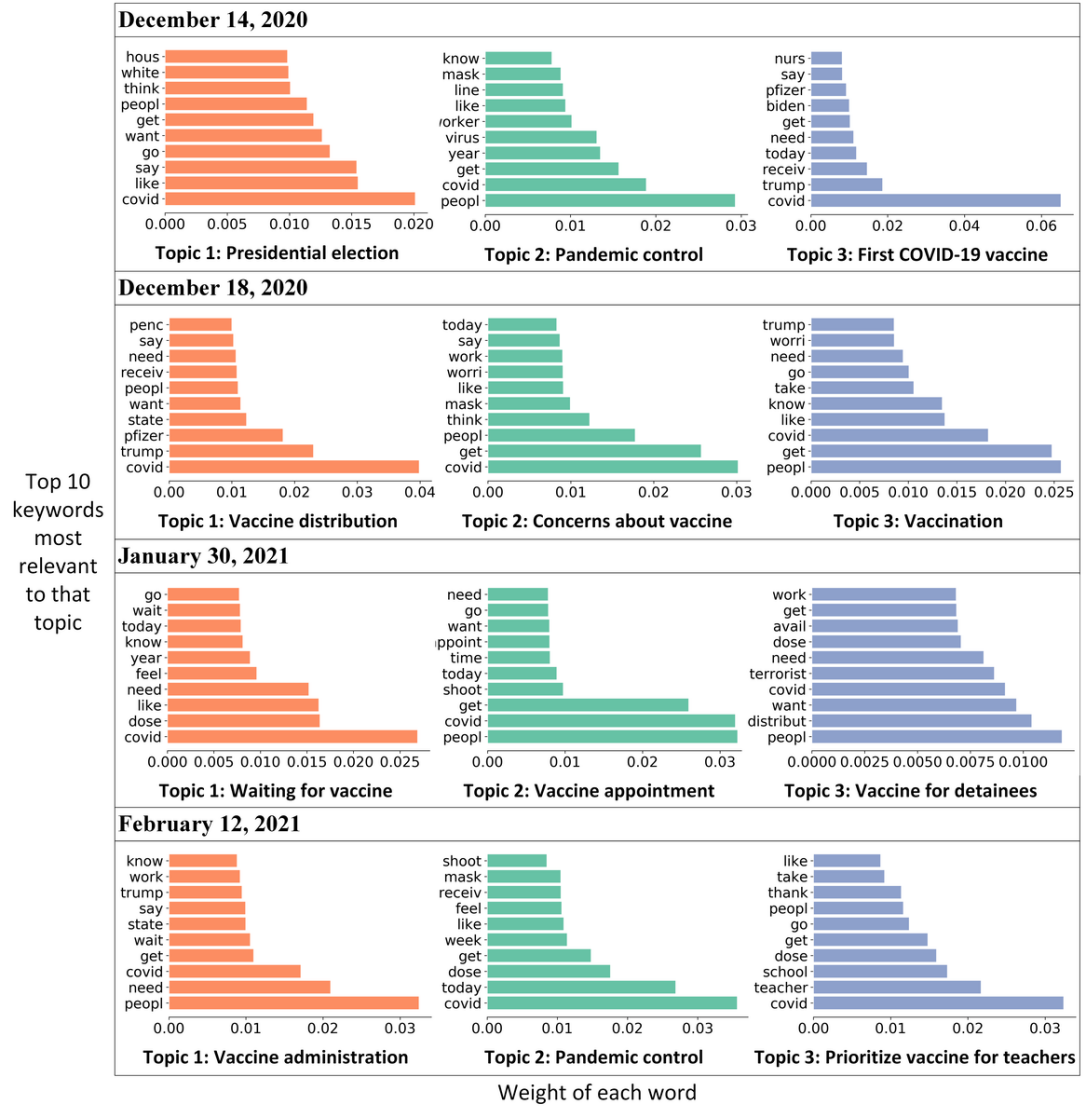
Three topics discussed on each of 4 key dates: December 14, 2020; December 18, 2020; January 30, 2021; and February 12, 2021.

Figure 1 shows the overall trends in the weekly sentiment scores, unveiling the increased positive attitude towards COVID-19 vaccines within the study period. We identified 11 key dates as turning points in sentiment scores or in the number of geotweets. Correspondingly, a total of 33 topics on these 11 key dates are summarized and presented in Figures 2-4. In Phase 1, changes in the sentiment score were relatively stable, except for a sharp drop on June 21, 2020. This drop could have resulted from the misinformation and conspiracy theories related to Bill Gates. Vaccine-adverse conspiracy related to Gates claimed that the pandemic is a cover for his plan to implant trackable microchips made by Microsoft [37]. Topic modeling suggests that Gates was referred to as “satan,” “terrorist,” and “evil” on that day (Figure 2).

In Phase 2, the first stimulus was observed on July 14, 2020, when the results of Phase 1 clinical trials by Moderna were published [35]. However, we did not observe a dramatic change in sentiment score until July 15, 2020, when Donald Trump tweeted “Great News on Vaccines!” [35]. Topic modeling suggests that keywords related to “good,” “trial,” “promis,” and “test” were widely discussed on July 15, 2020 (Figure 2). Speculation suggests that, compared to key events in the development of COVID-19 vaccines, comments from public figures on vaccination could trigger bigger changes in public sentiment.

Another sharp increase in sentiment score was observed on July 22, 2020, when the partnership between Pfizer and the US government accelerated the production and delivery of 100 million doses of COVID-19 vaccines [38]. The keywords “pfizer,” “govern,” and “million” were widely discussed and identified through topic modeling (Figure 3). On August 20, 2020, the sentiment score dropped dramatically after Kamala Harris formally accepted the Democrats’ vice-presidential nomination at the 2020 Democratic National Convention. Harris advocated, “There is no vaccine for racism,” mentioning the context of the racism protests for George Floyd and Breonna Taylor [39]. Of the keywords, “racism” and “kamala” were observed through topic modeling. Another increase in sentiment score appeared on November 9, 2020, when Pfizer announced that its vaccine is 90% effective (Figure 3) [40]. On the same day, Trump tweeted “STOCK MARKET UP BIG, VACCINE COMING SOON. REPORT 90% EFFECTIVE. SUCH GREAT NEWS!” Amid positive news from Pfizer, people questioned whether Pfizer purposefully released study results after Election Day, though Pfizer’s CEO claimed that the release timing had nothing to do with politics [41]. On that day, widely discussed keywords included “trump,” “pfizer,” and “elect” (Figure 3).

In Phase 3 on December 14, 2020, an increased sentiment score was observed when an intensive care unit nurse received the first COVID-19 vaccine in New York. On the same day, the Electoral College voted to cement Biden’s victory over Trump. Discussion regarding COVID-19 vaccines (“pfizer,” “nurs,” “receive”) quickly increased on Twitter, while other related discussions regarding mask wearing (“wear” and “mask”) and the presidential election (“house,” “trump,” “biden”) remained popular (Figure 4). By December 18, 2020, the sentiment score remained high as both Pfizer and Moderna were authorized for emergency use by the US Food and Drug Administration [42]. Trump tweeted “Moderna vaccine overwhelmingly approved. Distribution to start immediately.” Additionally, the fact that former Vice President Pence and second lady Karen Pence received a COVID-19 vaccine [43] was widely discussed (“penc” and “receiv”). Expectations for the COVID-19 vaccines were also discussed (“need” and “want”; Figure 4). On January 30, 2021, the Department of Defense paused a plan to give COVID-19 vaccines to detainees in the Guantanamo Bay prison camp [44], which raised queries of COVID-19 vaccine delivery, leading to a moderate decrease in the sentiment score. Keywords were observed, including “terrorist” and “distribut” through topic modeling (Figure 4). On February 12, 2021, an increased sentiment score was observed after the Biden administration announced the purchase of 200 million COVID-19 vaccine doses from Pfizer and Moderna [45]. Discussion surrounding the administration of COVID-19 vaccines was extensive (“wait,” “get,” “need”; Figure 4). Topic modeling also suggests that complaints were pervasive (“teacher,” “school,” and “get”; Figure 4) because teachers were not prioritized for vaccination in states despite the Center for Disease Control and Prevention’s recommendation.

**Figure 5:**
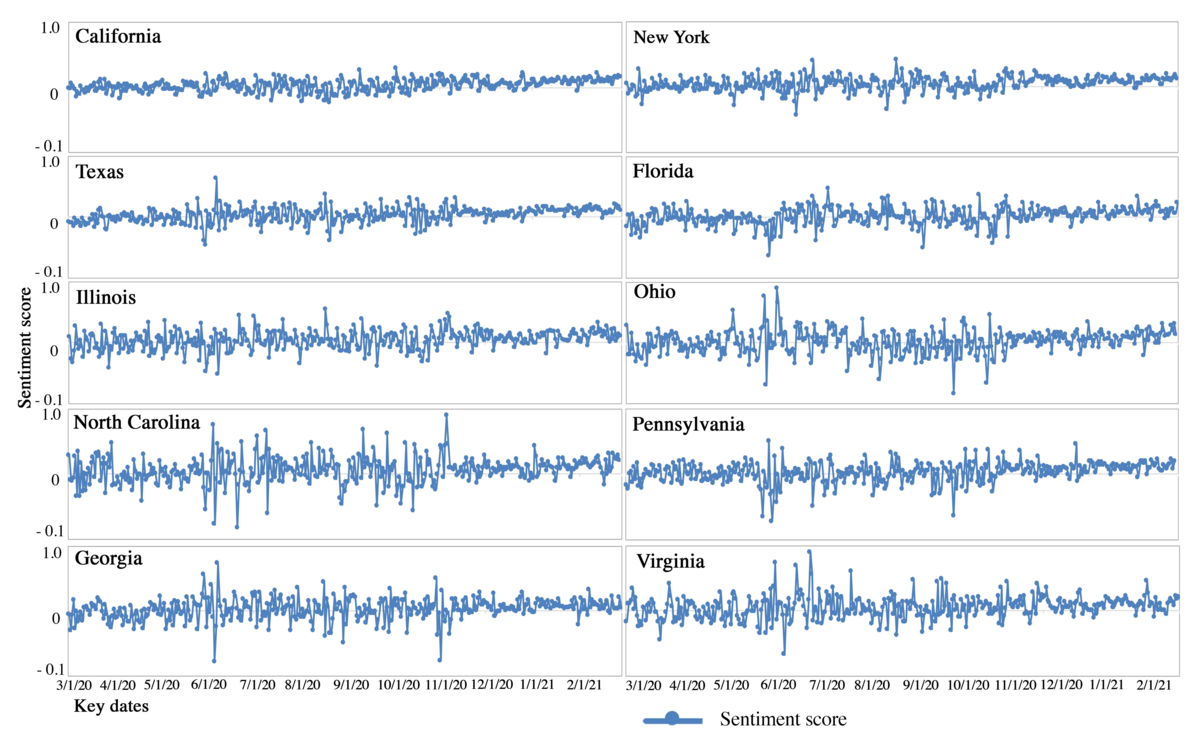
Sentiment scores in 10 selected states.

We then broke down the sentiment scores by state in tandem, along with the pandemic timeline. We present the results in the top 10 states with the largest number of geotweets (Figure 5), including California, New York, Texas, Florida, Illinois, Ohio, North Carolina, Pennsylvania, Georgia, and Virginia. The temporal patterns in sentiment scores vary across states, with more obvious fluctuations before November 2020 in Illinois, Ohio, North Carolina, Georgia, Pennsylvania, and Virginia. A number of sharp decreases in sentiment scores was observed in June 2020 in Illinois, North Carolina, Ohio, Pennsylvania, Georgia, and Virginia, in line with the tendency of sentiment drops at the national level. The states with relatively larger numbers of geotweets (ie, California, New York, Texas, and Florida) had more stable temporal trends and sentiment scores compared with the states with relatively smaller numbers of geotweets (eg, Ohio, North Carolina, Pennsylvania, Georgia, and Virginia).

**Figure 6:**
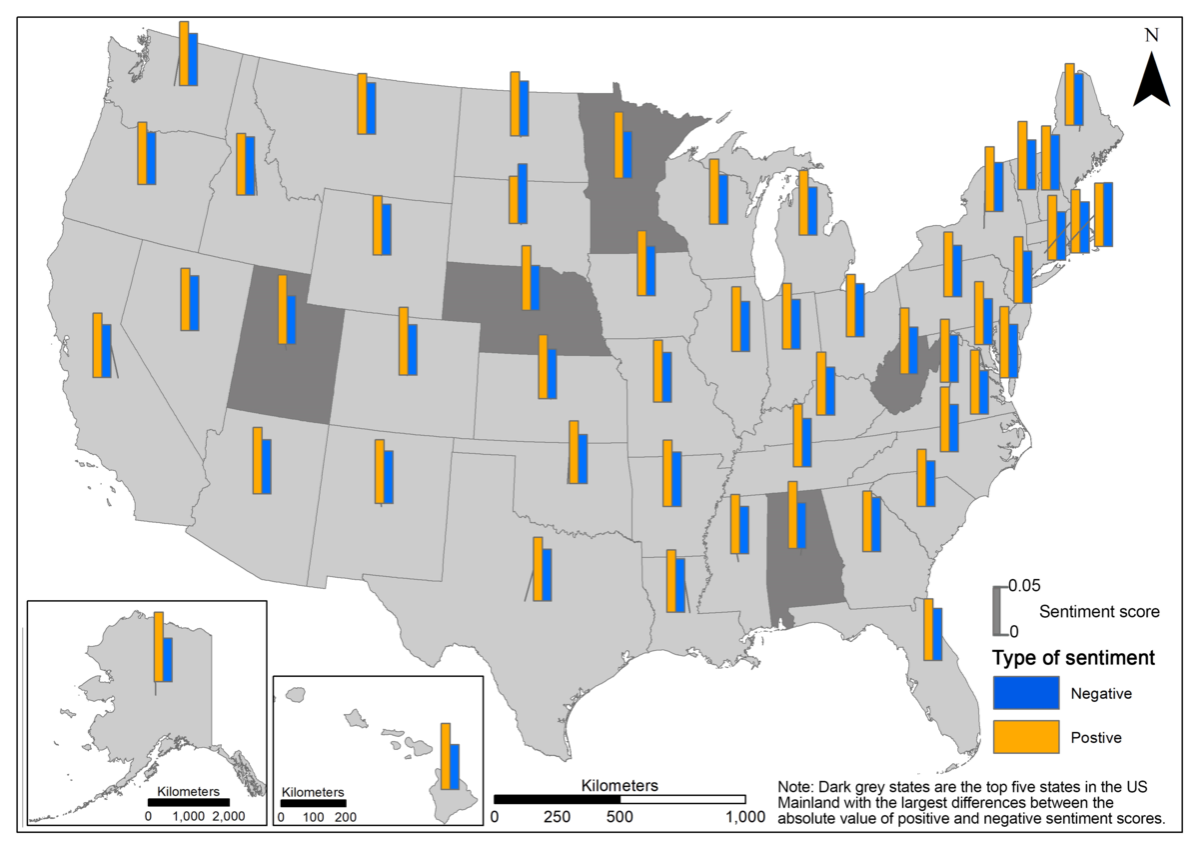
Absolute values of negative and positive sentiment scores at the state level.

We further examined the absolute values of the average positive and negative sentiment scores by states in Figure 6. In the majority of the states, the absolute positive sentiment score was larger than that of the negative sentiment score. The difference between the positive and negative sentiment scores was relatively more obvious in the mainland states of Alabama, Utah, Nebraska, Minnesota, and West Virginia (highlighted in dark grey in Figure 6), as well as in Hawaii and Alaska; the potential drivers triggering such differences across states may either relate to information or news spreading locally or be subject to the variations caused by the different sampling size in each state.

**Figure 7:**
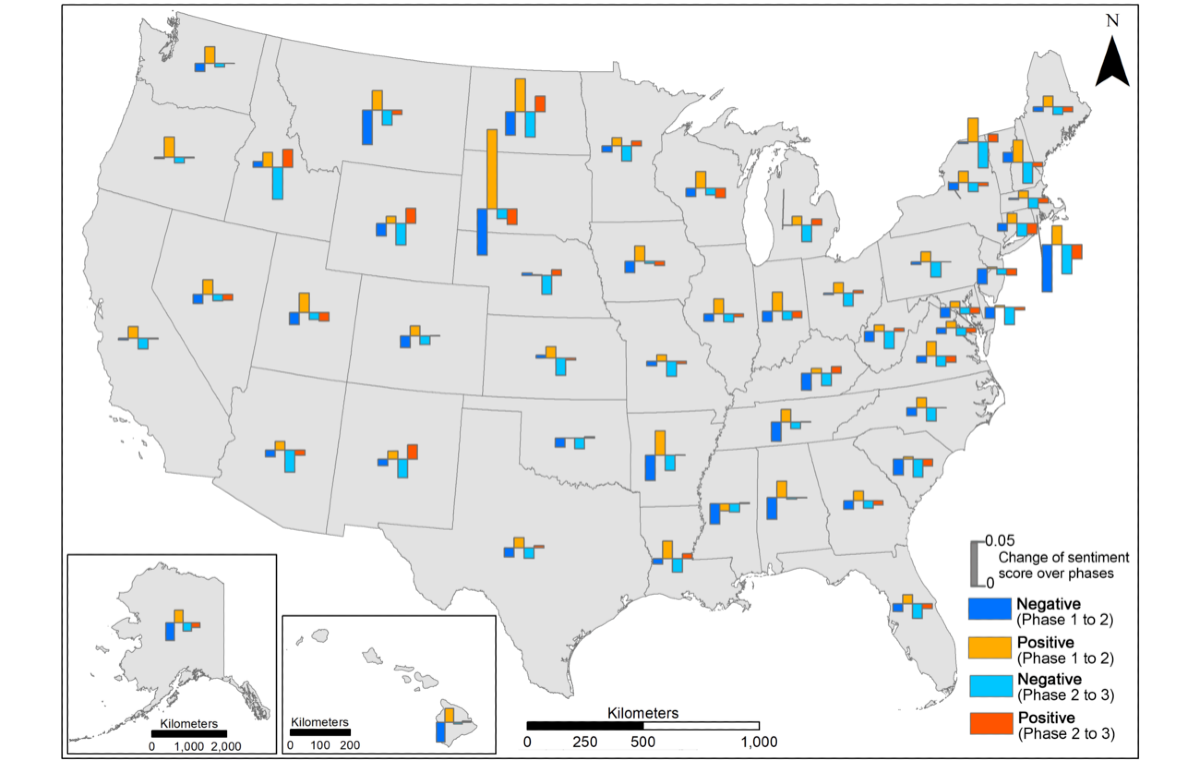
Changes in sentiment scores over the 3 phases at the state level.

The changes in positive and negative sentiment scores over 2 periods of time (Phase 1 to Phase 2; Phase 2 to Phase 3) were compared and are presented in Figure 7. From Phase 1 to Phase 2, an increase in positive sentiment scores (orange bars) appeared in most states, most obviously in South Dakota, followed by North Dakota and Arkansas; meanwhile, a decrease in negative sentiment scores (dark blue bars) was also observed in the majority of states, most obviously in South Dakota and Rhode Island, followed by Montana, North Dakota, and Arkansas. From Phase 2 to Phase 3, the decrease in negative sentiment scores (light blue bars) appearred in most states, most obviously in Idaho and Rhode Island, followed by North Dakota, Vermont, and New Hampshire. However, the change in positive sentiment scores (red bars) from Phase 2 to Phase 3 varied across states, with a slight increase that is more obviously observed in Idaho, North Dakota, and New Mexico, while a slight decrease is more obviously observed in South Dakota, Rhode Island, and Connecticut. In addition, the magnitude of both positive and negative sentiment scores from Phase 1 to Phase 2 (the height of dark blue and orange bars) was more obvious in most states than that of Phase 2 to Phase 3 (the height of light blue and red bars). This indicates that the fluctuation in people’s opinions towards vaccines became less obvious with the gradual development of vaccines and more encouraging news.

### Emotion Analysis

**Figure 8:**
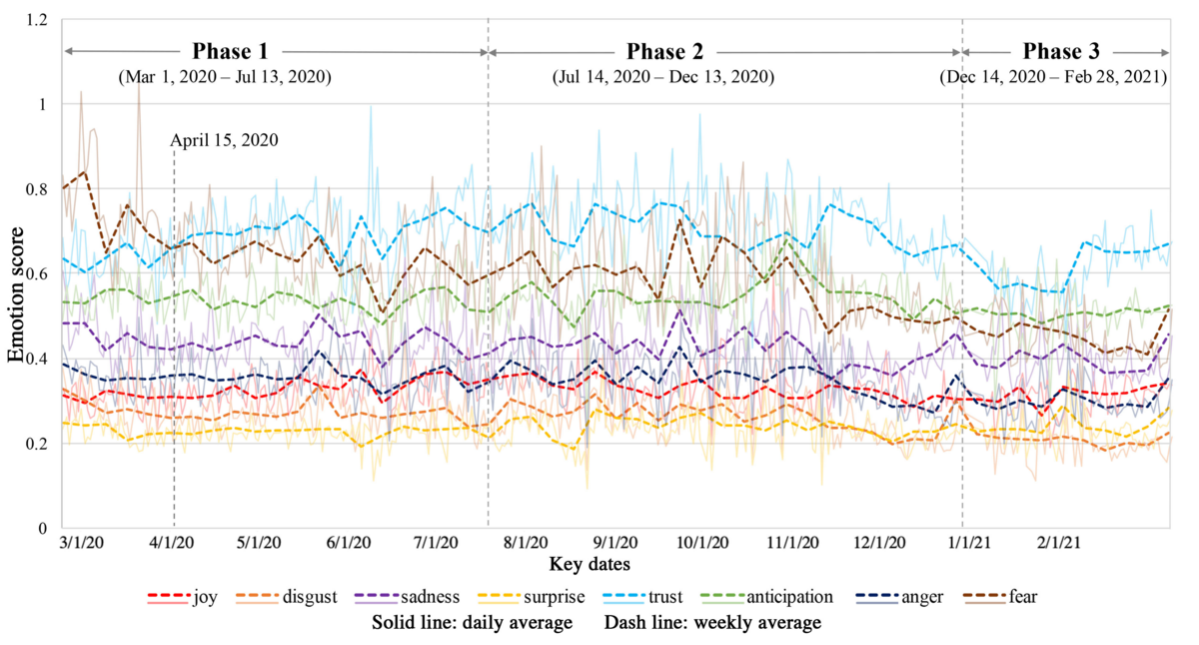
Average daily and weekly emotion scores over the entire study timeline at the national level.

Figure 8 shows the temporal patterns in the 8 types of emotion, including joy, trust, anticipation, trust, surprise, disgust, sadness, and fear. Through the vertical comparison of the weekly average trend lines (dashed lines), we found that the emotion with the highest weekly average scores along the majority of the timeline was trust (blue dashed line), followed by fear, anticipation, sadness, anger, joy, disgust, and surprise. It is worth noting that the weekly average emotion score of fear was higher than that of trust before mid April 2020, possibly due to rapid COVID-19 infection and ineffective control of viral spread at the early stage of the pandemic. These events may have caused fear, uncertainty, or even feelings of panic [46]. Although fluctuations in emotion scores (eg, local peaks and valleys) can be found within each of the 8 emotions, the general trend implies that the public’s trust in and anticipation towards vaccination were accompanied by a mixture of fear, sadness, and anger.

**Figure 9:**
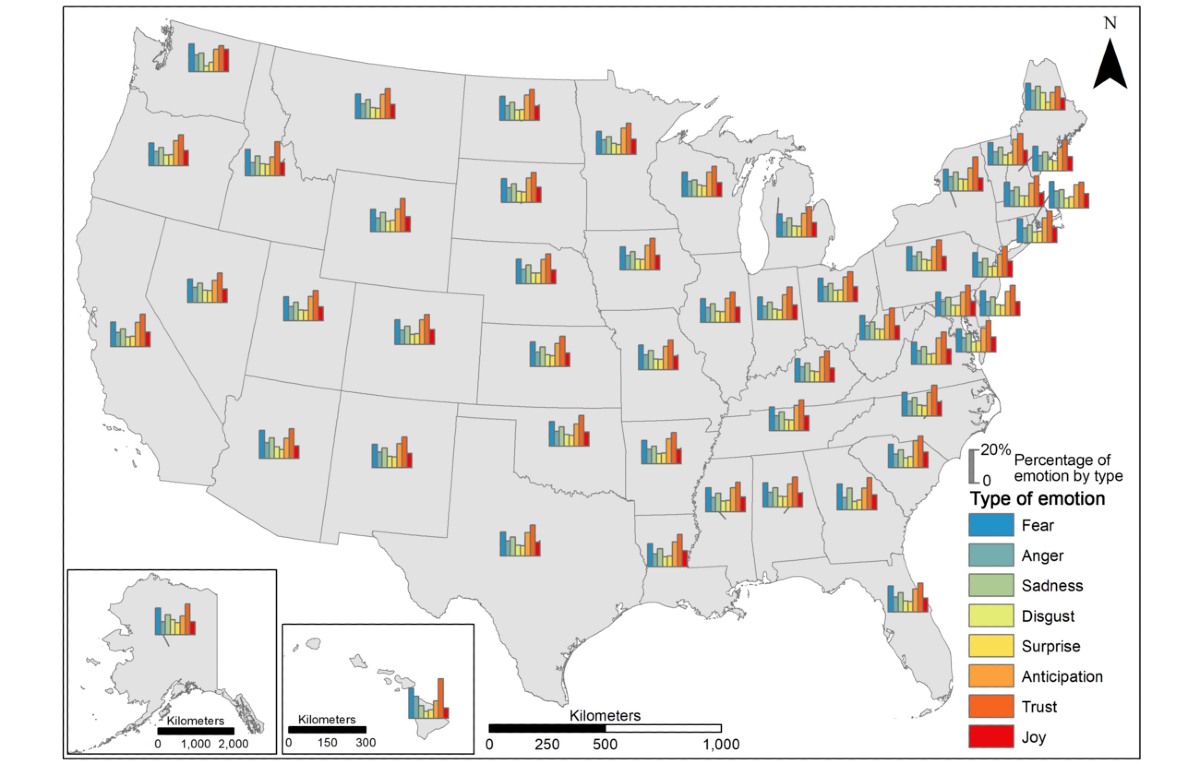
Percentage of 8 emotions expressed at the state level.

**Figure 10:**
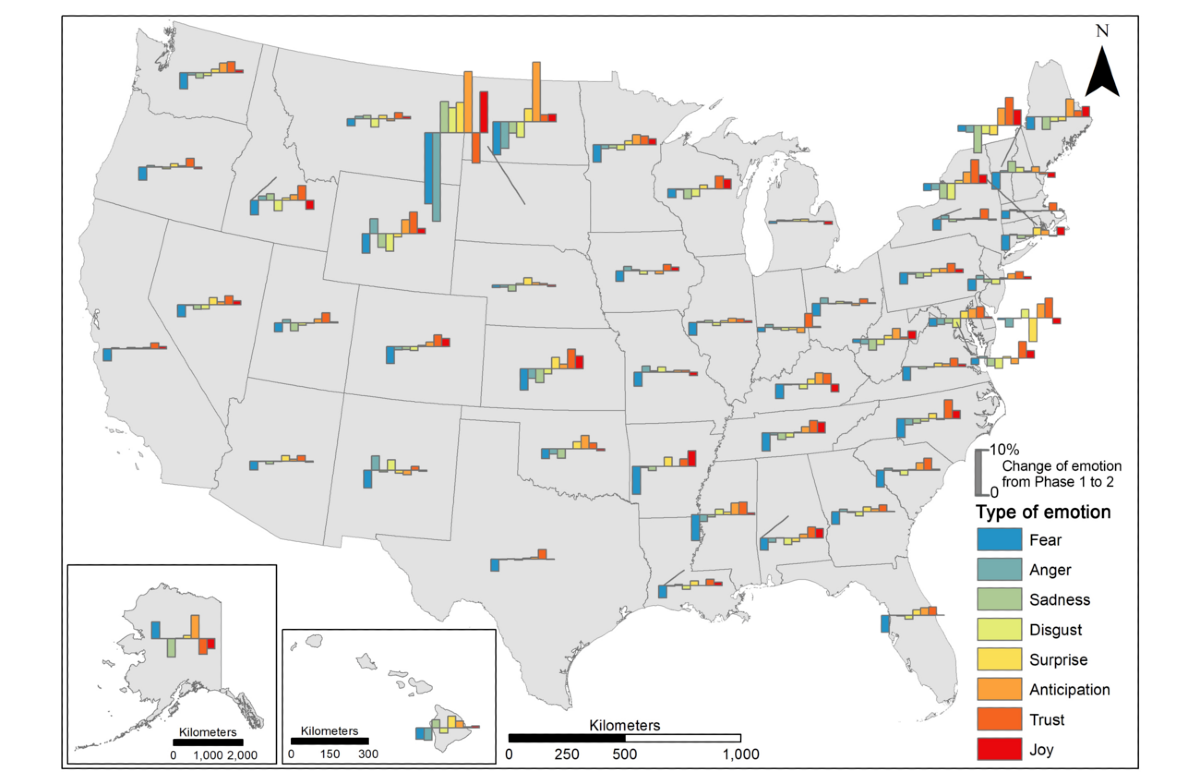
Change in emotions from Phase 1 to Phase 2 at the state level.

**Figure 11:**
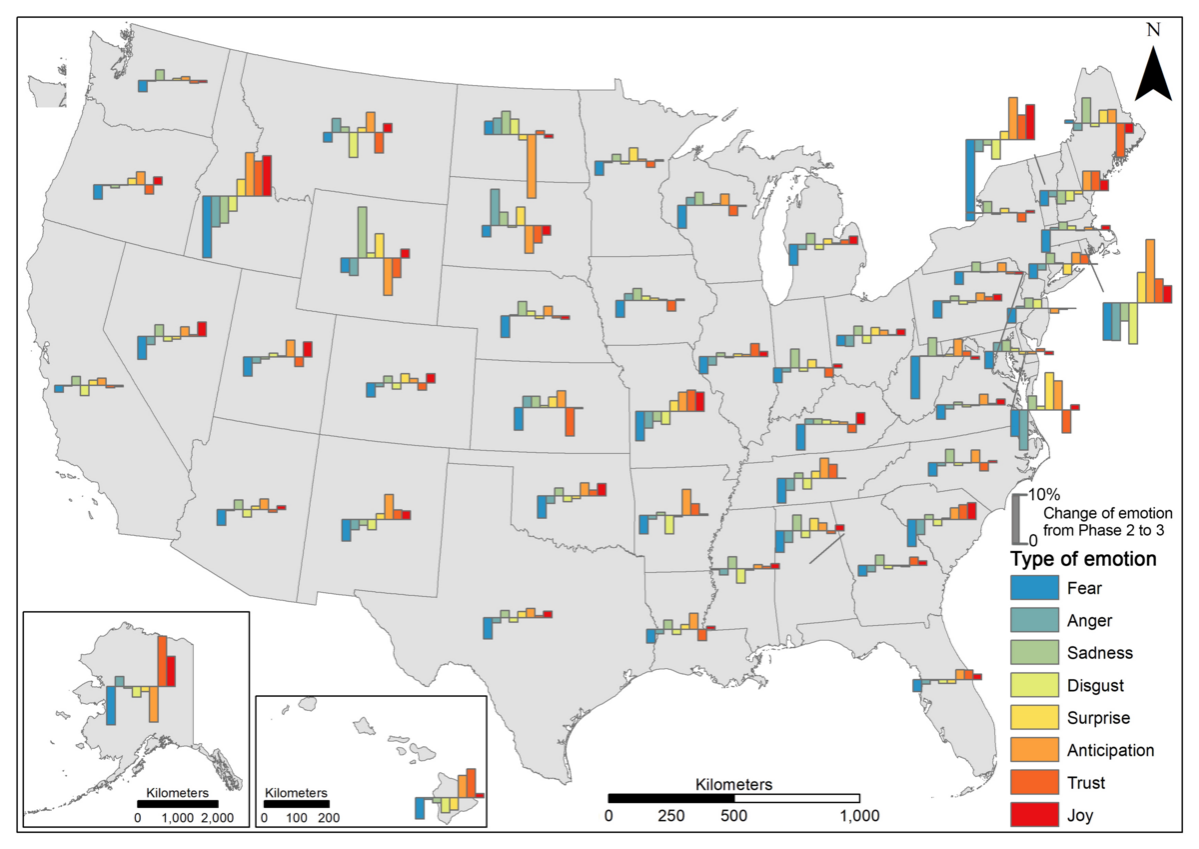
Changes in emotion from Phase 2 to Phase 3 at the state level.

We further investigated the relative distributions of 8 emotions in each state, as indicated by the percentage of emotion scores for each type with different colors (Figure 9). The overall patterns of the 8 emotions are consistent across most states. Throughout the entire timeline and in each of the 3 phases of the pandemic, trust was the dominant emotion towards vaccination over the full timeline of the pandemic. It was followed by anticipation, fear, sadness, anger, joy, disgust, and surprise. The state-level patterns largely align with the national pattern as depicted in Figure 9, although there are some exceptions, such as fear outweighing anticipation, joy, and trust (eg, Washington) and with fear, anger, and sadness outweighing other emotions (eg, Maine). As shown in Figures 10 and 11, the emotion of trust stayed consistent over time, while the changes in trends for other types of emotion were distinct across phases and by state.

We further compared the change in the percentage of emotions over 2 periods of time (Phase 1 to Phase 2; Phase 2 to Phase 3). From Phase 1 to Phase 2 (Figure 10), a decrease in fear (dark blue bars) was observed in most states, though its magnitude varied across states. This decrease was most obvious in South Dakota, followed by North Dakota, Arkansas, Mississippi, North Carolina, and South Carolina. The changes in anger, sadness, and disgust varied across states, with a general decrease in most states but sporadic increases in others (eg, Idaho, New Mexico, and New Hampshire). Furthermore, the combination of a decrease in fear and an increase in joy, trust, and anticipation was observed in most states except South Dakota. Throughout the period from Phase 2 to Phase 3 (Figure 11), it is difficult to generalize the pattern of emotion change across states in terms of type and magnitude. An increase in joy, trust, anticipation, and surprise along with a decrease in fear, anger, sadness, and disgust were the most notable (high bars) in Idaho and Rhode Island, followed by Missouri, Vermont, and New Hampshire. On the contrary, some states encountered a decrease in trust and anticipation in tandem with an increase in anger and sadness, including South Dakota, North Dakota, Montana, Kansas, Indiana, Maine, and Delaware. The complexity of emotion changes from Phase 2 to Phase 3 varied across states, reflecting the diversity in people’s opinions and psychological reactions to vaccination, which should be subject to an in-depth investigation of causality.

### Word Cloud Visualization

**Figure 12:**
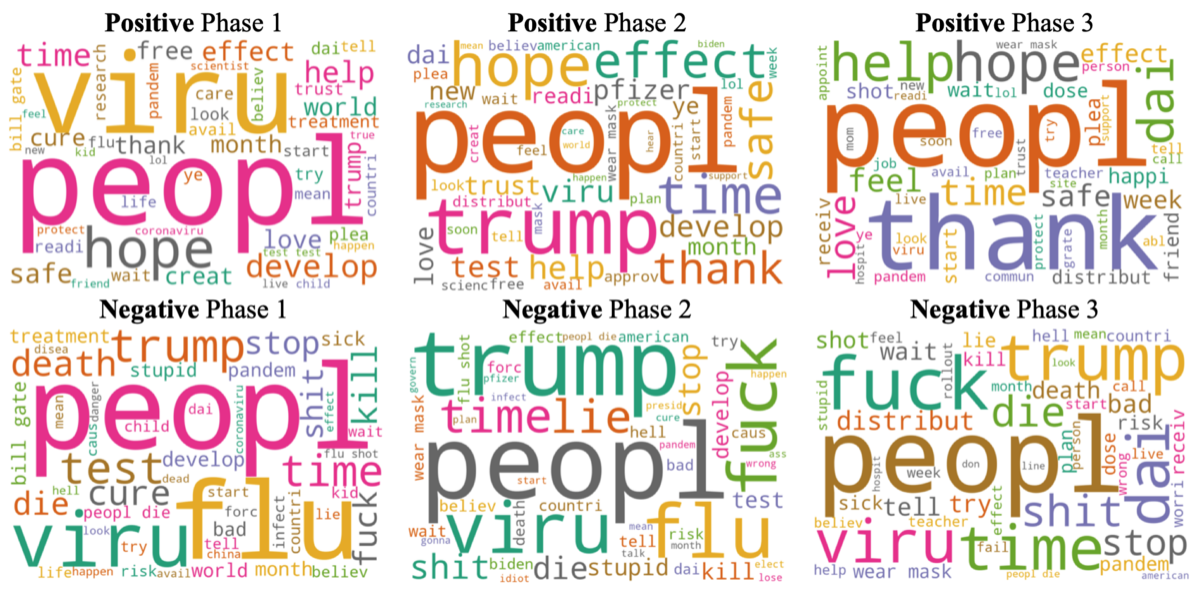
Popular keywords associated with positive and negative sentiments over 3 phases.

We produced word cloud mappings of 50 popular words associated with positive and negative sentiments over the 3 phases (Figure 12). The size of a word represents its popularity and the frequency with which it appears in tweets. Among the words associated with positive sentiment, the popular ones were “hope,” “help,” “thank,” “love,” “safe,” “cure,” and “free,” although the word “peopl,” with a more neutral nature, appears to be the most popular. Throughout the 3 phases, “hope,” “safe,” and “thank” grew larger from Phase 1 to Phase 3; in particular, “thank” became the most popular word in Phase 3. On the contrary, “flu,” “death,” “trump,” “fuck,” “lie,” “die,” “kill,” “shit,” and “stupid” were popular words associated with negative sentiment. Over the 3 phases, “flu” became smaller from Phase 1 to Phase 3 whereas “die,” “fuck,” “shit,” and “trump” evolved to be larger from Phase 1 to Phase 3; in particular, “trump” became predominant in Phase 2 possibly due to Trump’s increasing popularity caused by the 2020 Presidential Election. More specifically, while people were waiting for the news of COVID-19 vaccine development during Phase 1, their uncertainties on potential vaccines were reflected in the included keywords, which were related to the coronavirus and public’s frustration of the pandemic (eg, “viru,” “death,” “cure,” and “test”). Some keywords related to the COVID-19 vaccine were also observed, including “hope” and “develop.” Positive news about the development of COVID-19 vaccines appeared in Phase 2, which brought hope as well as misinformation regarding the vaccines to the public. At this stage, more specific information about COVID-19 vaccines was discussed (eg, “Pfizer,” “effect,” “risk,” “develop,” and “approve”), as compared to Phase 1. With Pfizer and Moderna vaccines approved during Phase 3, the public’s attention moved from vaccine development towards vaccine distribution (“distribution,” “wait,” and “free”), effectiveness (“safe” and “risk”), and priority (“teacher”). In all 3 phases, public figures (eg, “Trump,” “Biden,” and “Bill Gates”) contributed to hot topics with impacts on both positive and negative sentiments.

## Discussion

### Principal Findings

Drawing on geotweets from March 1, 2020 to February 28, 2021, this study examined public opinion on COVID-19 vaccines in the United States, by unveiling the spatiotemporal patterns of public sentiment and emotion over time, modeling the popular keywords and topics of Twitter contents, and analyzing the potential drivers of public opinion on vaccines. Our findings indicate that critical social or international events or announcements by political leaders and authorities may have potential impacts on public opinion towards COVID-19 vaccines. Such examples include the vaccine-adverse conspiracy related to Bill Gates on June 21, 2020, the tweet by Donald Trump of “Great News on Vaccines!” on July 14, 2020, Kamala Harris’s advocacy of “There is no vaccine for racism” on August 20, 2020, Biden’s victory of the presidential election over Trump on December 14, 2020, and the authorized emergent usage of Pfizer and Moderna on December 18, 2020. In the proposed 3 phases over the study timeframe, changes in public opinions on vaccines varied across space and time. More specifically, the fluctuation in people’s sentimental response to the vaccine during the earlier stage of the pandemic was more obvious compared to that in the later stage of the pandemic. However, an increase in positive sentiment in parallel with a decrease in negative sentiment were generally observed in most states, reflecting the rising confidence and anticipation of the public towards COVID-19 vaccines. Furthermore, the public’s 8 types of emotion towards the COVID-19 vaccine displayed a general trend of a combination of trust and anticipation with a mixture of fear, sadness, and anger. Moreover, the word cloud mapping showed that positive keywords including “hope,” “safe,” and “thank” grew larger from Phase 1 to Phase 3; in particular, “thank” became the most popular word in Phase 3, indicating the public’s increasingly positive response towards vaccination. In all 3 phases, public figures (eg, “Trump,” “Biden,” and “Bill Gates”) contributed to the most popular topics, impacting both positive and negative sentiments. The aforementioned findings reveal the diversity and complexity of people’s perception on and their psychological reaction towards COVID-19 vaccines, which indicates a further need to be cautious in the interpretation of analytical outcomes and to initiate an additional in-depth investigation of the causality.

Our findings are partially supported by the current literature. Hussain et al [14] observed a marked increase in positive sentiment toward COVID-19 vaccines in the United States from March 1, 2020 to November 22, 2020. Guntuku et al [18] and Roy and Ghosh [47] found that Republican legislators became more engaged in public discussion on vaccine progress, which may have implications for COVID-19 vaccine uptake among their followers. Germani and Biller-Andorno [17] revealed that antivaccination supporters have been heavily engaged in discussions and dissemination of misinformation and conspiracy theories. Considering the limitations (ie, random sample) inherent in Twitter data, it is important to propose alternative data that provide a complementary understanding of public opinions towards the COVID-19 vaccine to promote vaccination in the United States.

### Implications and Recommendations

The emergence of the internet and social media has provided new platforms for persuasion and the rapid spread of (mis)information, which leads to new opportunities for and challenges to the communication of vaccine information [48]. There are over 4.3 billion people using the internet nowadays, with 3.8 billion of these individuals as social media users [49]. The popularity of social media platforms coupled with the advent of digital detection strategies benefit public health authorities by enabling the monitoring of public sentiment towards vaccine-relevant information in a geo-aware, (near) real-time manner. This can inform more effective policymaking and promote participatory dialogue to establish confidence towards vaccines, in order to maximize vaccine uptake. Some of our findings add new value to the current scholarship and also provide new insights and suggestions for policy implications with regard to safeguarding societal and economic health.

First, our findings indicate that public figures, especially politicians, play a crucial role in impacting the public’s opinions on vaccination. Negative opinions expressed by public figures about a vaccine could impact a large population of people, especially those who do not hold an unswayable opinion [48]. People tend to believe public figures’ opinions, as they are elected officials who can influence health care systems and are perceived to have more information about a vaccine [50,51]. Thus, public figures have a responsibility to disseminate accurate health information and should be cautious in expressing their opinions in public. This also highlights the necessity of considering the impact that public figures within vaccine campaigns have on upholding the public’s confidence towards the concept of vaccination.

Second, our study reveals that vaccine-adverse conspiracy theories led to a sharp decline in sentiment scores. We need to be aware of the fact that social media platforms with a massive number of users, to some degree, “disrupted” traditional vaccine information communication [52], allowing antivaccination advocates to disseminate misleading messages to a certain audience, whose views on vaccination could be susceptible to change. However, it also means that governmental officials should consider using these platforms to communicate with individuals directly about vaccination via geotailored messages to address concerns specific to a certain region.

Third, different states demonstrated various trends in sentimental and emotional scores. Our geospatial analysis and map visualization [53] better portray more aspects of users’ attitudes towards COVID-19 vaccines. This helps identify the areas with high negative sentimental and emotional scores that require further research to understand the public's underlying fears and concerns about COVID-19 vaccines. We also recommend government and public health agencies conduct COVID-19 vaccine campaigns in these areas to address people’s fears and concerns about COVID-19 vaccines and provide guidance to access available vaccines.

### Limitations and Future Work

Our study has several limitations that can be improved in future studies. First, the demographics of Twitter users is typically characterized by younger users who are avid users of mobile phone apps and the internet, and such users may not be able to reflect the opinion and perception of the general public with varying demographics and socioeconomic statuses [54,55]. In addition, the representativeness of Twitter users is not stationary but geographically varying [56,57]. Like other studies that rely on digital devices, the “digital divide” [58] issue needs to be acknowledged. This study only accounts for the reactions from Twitter users to vaccines, which, to some degree, neglect the underprivileged members of society (especially the poor and elderly), inhabitants of rural areas (who do not have access to digital devices), and those who are not willing to share their thoughts on social media platforms. Additionally, the Twitter API that we used allows access to approximately only 1% of the total records [59]. As Padilla et al [60] demonstrated, tweet sentiment can be impacted based on attraction visits throughout the course of a day. Hence, future work needs to increase the sample size to reduce the uncertainties and fluctuations of sentiment scores and emotions. Efforts are also needed to distinguish between local residents and visitors and also conduct investigations under finer temporal scales. In early 2021, Twitter released a new Twitter API (academic research product track) that grants free access to a full-archive search with enhanced features and functionality for researchers to obtain more precise, complete, and unbiased data for analyzing the public conversation [61]. Further efforts can be made to explore the potential of this new API in mining public opinions towards COVID-19 vaccines at a more granular scale. Since emotion is a complex and integrated product of human feelings [62], future research efforts can be put into exploring more diverse dimensions of emotion, on top of the 8 primary types of emotion. Moreover, disaster and crisis management includes 4 phases, namely prevention (capacity building), preparation (early warning), response (search, rescue, and emergency relief), and recovery (rehabilitation) [63]. Management of the COVID-19 pandemic is still in the response phase. For policy and decision-making endeavors that are pertinent to COVID-19 crisis management, it will be highly beneficial if researchers and practitioners continuously monitor emotional and perspective variations throughout the response and also extend the study timeline to the recovery phase or massive vaccination phase in the post-pandemic years. More importantly, to understand the impact of vaccination on countries, the workflow and methodology used in this study can be applied in multiple languages to global-scale geotweets.

## Abbreviations

API: application programming interface
LDA: Latent Dirichlet Allocation
NRCLex: National Research Council Canada Lexicon
VADER: Valence Aware Dictionary for Sentiment Reasoning
